# Improving taste sensitivity in healthy adults using taste recall training: a randomized controlled trial

**DOI:** 10.1038/s41598-022-18255-z

**Published:** 2022-08-16

**Authors:** Yuta Otsubo, Midori Miyagi, Hideki Sekiya, Osamu Kano, Satoru Ebihara

**Affiliations:** 1 Department of Rehabilitation Medicine, Toho University Graduate School of Medicine, Tokyo, Japan; 2 Department of Oral Surgery, Toho University Omori Medical Center, Tokyo, Japan; 3 Department of Neurology, Toho University Omori Medical Center, Tokyo, Japan; 4 Department of Internal Medicine and Rehabilitation Science, Tohoku University Graduate School of Medicine, Seiryo-machi 1-1, Aoba-ku, Sendai, 980-8574 Japan

**Keywords:** Neurophysiology, Nutrition, Rehabilitation, Preclinical research

## Abstract

Although many patients suffer from taste disorder, methods to improve taste sensitivity are limited. To develop a taste recall training method to improve the perception of taste, 42 healthy individuals were randomly assigned to either the training or the control group. Using the filter paper disc method, participants in the training group were asked to match the four tastes (sweetness, saltiness, sourness, and bitterness) between those of taste recognition thresholds and those of a one-step higher concentration until they get them right. Then, they were asked to match the four tastes between those of one-step lower and one-step higher in concentration from their taste recognition thresholds until they get them right. Finally, they were asked to match the four tastes between those of one-step lower concentration and those of their taste recognition thresholds until they get them right. This training was repeated until perfectly matched. The taste recall training program led to a lowered taste recognition threshold in healthy adults for each taste quality, suggesting the improvement of taste sensitivity. This lowered threshold for each taste was observed with each additional training session. We conclude that this taste recall training method might be a therapeutic approach for treating taste disorder.

## Introduction

Taste is an important sensory attribute for determining the safety of what we eat. Taste has been linked to the nutritional content of food and is one of the primary factors affecting people’s daily eating behavior^1^. Taste disorders may result from lesions in taste receptors or from the peripheral and central nervous system. Causes for this syndrome include zinc deficiency, oral diseases, systemic diseases (e.g., malignancies), drug-related side effects, head and facial trauma, psychogenic factors, and aging. However, taste disorders can also be idiopathic, that is, of unknown origin. Younger and older people alike may be affected by this condition since its causes are so diverse. Prior studies have reported that aging causes a reduction in the number and size of taste buds^2^ as well as a decrease in the central neural responses^3^. Additionally, because multiple comorbidities are prevalent among older adults, they are more likely to develop taste disorders. Therefore, an aging population is highly likely to be affected by this condition.

Taste disorder has been recently identified as a symptom of COVID-19. Following the outbreak of the COVID-19 pandemic, the number of people affected by taste disorder has rapidly increased^4^. Taste disorder in older adults has been shown to correlate strongly with a reduced QOL^5^ and should not be taken lightly.

However, while the number of patients affected by taste disorder is expected to increase, only symptomatic treatments have been established for this condition. The treatments include zinc replacement treatment for zinc deficiency, treatment for oral diseases such as oral candida and dry mouth, and discontinuation of drugs in cases of drug-induced taste disorder. However, many patients do not show improvement even after symptomatic treatment. Given the lack of specific treatments targeting the underlying etiological (e.g., age-related, idiopathic, etc.) factors of the disease, a considerable number of patients chronically suffer from this condition^6^. Moreover, taste disorder occurring after radiation therapy for cancer of the head and neck and after systemic chemotherapy for other malignancies can be highly refractory to treatment^7^.

Similar to taste disorder, olfactory impairment is a sensory disturbance linked to food consumption and is generally treated symptomatically. However, olfactory training, recently reported to have been effective in treating olfactory impairment^8^, is gaining attention as an alternative therapeutic approach in cases where symptomatic treatment has not been sufficiently effective. Classic olfactory training consists of having patients with taste disorder smell four different odors for several months^9^. Recent studies have elucidated the effect of exposure to a dozen of different odors as well as odors at different concentration levels, leading to improvements in olfactory training methods^10^. A common characteristic of these training methods is the repeated exposure of olfactory-impaired patients to several kinds of odors.

Based on the abovementioned olfactory training techniques, we developed a taste recall training method using filter paper discs as a new therapeutic approach for treating taste disorder. The objective of this study was to test the effectiveness of this training method on healthy individuals before applying it to patients with taste disorders.

## Results

All 42 participants (males: 26, females: 16, age: 27.5 ± 3.7 years) enrolled in the study had good oral health conditions as assessed using the OHAT-J and the Eichner classification. Participants were randomly assigned to either the training group or control group, each group had 21 participants. The first potential participant was screened on 22/09/2021, and the last participant’s final visit was on 26/01/2022. The trial ended after the last randomized participant completed the study. No adverse effects or abnormalities were observed in any of the participants. As shown in Table 1, no significant difference was observed between the control and training groups in terms of age, gender, BMI, oral moisture status as measured using an oral moisture checking device, and tongue pressure as measured using a tongue pressure measuring device. At the initial sensitivity evaluation for each of the four tastes (sweetness, saltiness, sourness, and bitterness), no significant difference was observed between the two groups (Table 1).

**Table 1:** Comparisons of baseline characteristics between control and training groups.

	Control	Training	P-value
Total participants, n	21	21	
Sex, male, n (%)	15 (71.4)	11 (52.4)	0.34
Age, mean (SD)	26.81 (2.96)	28.10 (4.30)	0.27
BMI, kg/m^2^, mean (SD)	21.79 (2.55)	22.05 (2.57)	0.75
Oral moisture degree, mean (SD)	29.06 (1.78)	30.13 (3.40)	0.21
Tongue pressure, kPa, mean (SD)	38.21 (9.38)	34.05 (9.28)	0.16
**Taste sensitivity**			
Sweetness (sucrose)	2.19 (0.51)	2.19 (0.75)	1.00
Saltiness (NaCl)	1.86 (0.65)	2.00 (0.95)	0.57
Sourness (tartaric acid)	2.33 (0.58)	2.48 (0.60)	0.44
Bitterness (quinine HCl)	2.05 (0.59)	2.24 (0.77)	0.37

No significant difference was observed between the two groups in gender, age, BMI, oral moisture content, and tongue pressure. Additionally, no significant differences were observed in the initial taste recognition thresholds for all taste qualities (sweetness, saltiness, sourness, and bitterness) between the two groups.

*SD* standard deviation, *BMI* body mass index.

**Figure 1:**
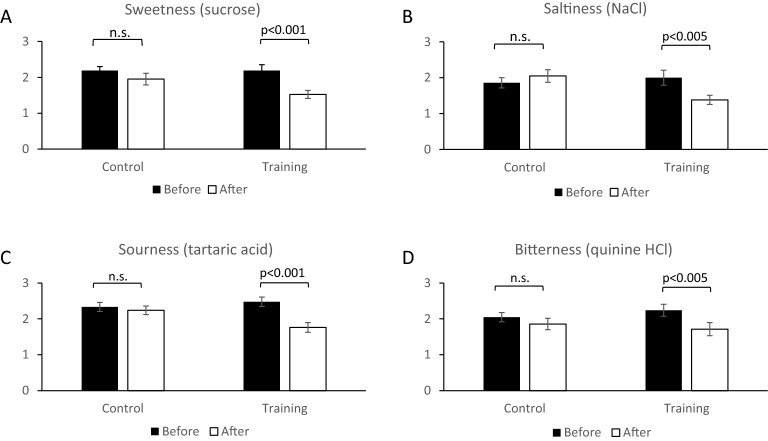
Initial and final taste recognition thresholds and their changes in the control and training groups. (**A**) Sweetness (sucrose); initial and final mean in the control and training groups were 2.19 ± 0.11 to 1.95 ± 0.16 and 2.19 ± 0.16 to 1.52 ± 0.11 (P < 0.001), respectively. (**B**) Saltiness (NaCl); initial and final mean in the control and training groups were 1.86 ± 0.14 to 2.05 ± 0.18 and 2.0 ± 0.20 to 1.38 ± 0.13 (P < 0.005), respectively. (**C**) Sourness (tartaric acid); initial and final mean in the control and training groups were 2.33 ± 0.13 to 2.24 ± 0.12 and 2.48 ± 0.13 to 1.76 ± 0.14 (P < 0.001). (**D**) Bitterness (quinine HCl); initial and final mean in the control and training groups were 2.04 ± 0.13 to 1.85 ± 0.16 and 2.24 ± 0.17 to 1.71 ± 0.18 (P < 0.005). Initial (black bar) and final (white bar) taste thresholds are indicated as mean ± SD. P values were calculated using paired-student t-test. *n.s.* denotes not significance.

The mean variation between the initial and final taste sensitivity evaluation in the control group was 2.19 ± 0.11 to 1.95 ± 0.16 for sweetness, 1.86 ± 0.14 to 2.05 ± 0.18 for saltiness, 2.33 ± 0.13 to 2.24 ± 0.12 for sourness, and 2.04 ± 0.13 to 1.85 ± 0.16 for bitterness indicating no significant changes. The mean variation between the initial and final taste sensitivity evaluation in the training group was 2.19 ± 0.16 to 1.52 ± 0.11 (P  < 0.001) for sweetness, 2.0 ± 0.20 to 1.38 ± 0.13 (P < 0.05) for saltiness, 2.48 ± 0.13 to 1.76 ± 0.14 (P <  0.001) for sourness, and 2.24 ± 0.17 to 1.71 ± 0.18 (P <  0.005) for bitterness, indicating significant improvements (Fig. 1).

All participants were interviewed to determine adverse effects after the final taste sensitivity evaluation and none were reported.

**Figure 2:**
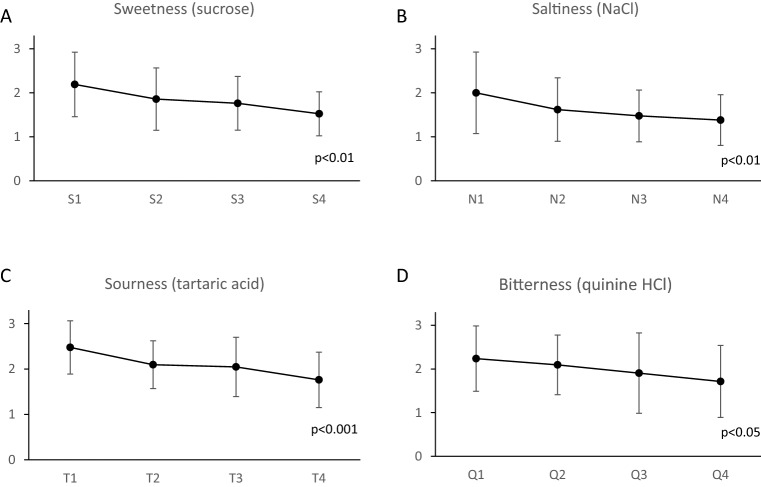
Daily changes in taste recognition thresholds of the training group. Daily changes in taste recognition thresholds of the training group with respect to (**A**) sweetness, (**B**) saltiness, (**C**) sourness, and (**D**) bitterness. The data represent mean ± SD. S: sweetness, N: saltiness, T: sourness, Q: bitterness. S1, N1, T1 and Q1 represent taste recognition thresholds on day 1, S2, N2, T2 and Q2 represent taste recognition thresholds on day 2, S3, N3, T3 and Q3 represent taste recognition thresholds on day 3 and S4, N4, T4 and Q4 represent taste recognition thresholds on day 4. P values were calculated using a Friedman test.

Figure 2 shows the changes in taste recognition thresholds before each training session for the 21 participants in the training group. Decreasing values of taste thresholds for each taste were observed with each additional training session, and the results of the Friedman test also showed significant differences for each taste.

## Discussion

The results of the study showed that our taste recall training program led to a decrease in the value of taste recognition thresholds in healthy adults for each taste quality (sweetness, saltiness, sourness, and bitterness), suggesting an increase in sensitivity of taste perception.

While the underlying causes of taste disorder are diverse, this condition has been recently reported as a post COVID-19 symptom. Goërtz et al. have reported cases showing the persistence of taste disorder for up to 79 days following COVID-19 infection^11^. A higher incidence of taste disorder has also been reported in older adults^12^. Therefore, the number of patients affected by this condition is expected to increase progressively worldwide. Moreover, treatment for taste disorder is symptomatic and, in some cases, does not yield satisfactory results^13^. The taste recall training program proposed in this study significantly increased the sensitivity of taste perception in healthy adults and may alleviate taste disorder in cases where no effective treatment is available. The method of administering the taste solutions is highly safe. This was also supported by the fact that the participants reported no side effects during and after the training. Further studies are needed to investigate whether the combined use of taste recall training and symptomatic treatments leads to greater improvement in taste sensitivity and shorter treatment duration.

Many prior studies on the topic of olfactory training have reported the good results obtained through this treatment. Standard olfactory training consists of having patients with olfactory impairment smell four different kinds of odors for a few months^14^; however, in recent years, methods using a dozen or more odors as well as odors at different concentration levels have been tested^15^. A common characteristic of all these training methods is that they repeatedly expose patients with olfactory impairments to several kinds of odors. The taste recall training method proposed in this study was developed based on the olfactory training method. In fact, the process of having the participants repeatedly exposed to four different taste qualities is very similar to that described in olfactory training.

The study’s findings suggested that participants’ exposure to each taste at lower concentration levels may reduce taste recognition thresholds resulting in increased sensitivity of taste perception. These findings are based on the premise that a specific taste may be perceived even at concentration levels that are lower than the taste recognition threshold owing to the brain’s capacity to elaborate vague gustatory stimuli into a specific taste perception. Prior studies employing the filter paper disc method reported that patients with taste disorder have elevated taste recognition thresholds^16,17^. The taste recall training method devised in this study is expected to effectively alleviate this condition by lowering taste recognition thresholds.

Taste disorders include hypogeusia (difficulty in perceiving taste qualities), ageusia (complete inability to perceive any taste quality), dissociated taste disorder (inability to perceive only specific taste qualities), and heterogeusia (inappropriate perception of taste quality). Research on the anatomy and physiology of taste cells and receptors in relation to taste perception as well as research on pathophysiology of taste disorders has been progressing steadily^18^. Currently, the types of odors used in olfactory training vary and are not standardized. Furthermore, reagents used in the taste recall training method presented in this study were prepared for testing rather than for training purposes but are commonly used in daily clinical practice and are readily available. In the future, we hope that further validation of the method’s effectiveness, promoted by the accumulation of knowledge obtained through its widespread use, may allow the targeting of various categories of taste disorders.

The effectiveness of neuroplasticity induced by repetitive transcranial magnetic stimulation therapy has been recently reported to be a major factor in the treatment of neurological disorders^19^. Likewise, neuroplasticity is indicated as the mechanism of action in olfactory training for olfactory impairment. In a study that used mice models, Kikuta et al. reported that after the artificial injury of olfactory cells in mice, which were unilaterally deprived of olfactory sensory input by inserting nasal plugs in their nostrils, a higher regeneration capacity of olfactory cells was observed in the olfactory epithelium of the nondeprived side compared to the olfactory epithelium of the deprived side^20^. Kollndorfer et al. used fMRI to examine the activity of neuronal networks linking olfactory-related brain areas in patients with olfactory dysfunctions who underwent olfactory training. Study results showed an increase in activity of such networks following olfactory training^21^. The above findings suggest that olfactory training may act on both peripheral plasticity at the cellular level and central neuronal networks. Olfactory and gustatory sensory information are received from peripheral receptors and transmitted via the hypothalamus to the hippocampus, the amygdala, and, eventually, to the olfactory and gustatory areas^22,23^. Because of the structural similarity of transmission pathways, taste recall training, like olfactory training, may act on peripheral cells and central neuronal networks, and can improve with the effect of neuroplasticity. Future studies should focus on the differences in brain activity and changes in taste buds in patients with taste disorders before and after taste recall training.

It is also necessary to clarify that taste and smell have different relationships with human memory. Olfactory has been reported to be more closely related to memory than taste^24^. Additionally, while olfaction can detect an object from an odor, taste identifies the tastes of various raw materials as similar taste qualities^25,26^. Moreover, while humans have about 400 odorant receptor genes, there is almost one type of sensor for sweetness, umami, sourness, saltiness, and about 25 types of sensors for bitterness^27,28^. Since taste is thus identified by a small number of types of sensors on the surface of the tongue, it is difficult for the tongue to identify a huge variety of foods in detail with such a small number of sensors.

This study has several limitations. First, the taste recall training method used in this study was tested on healthy adults, and it is unclear whether it may be truly effective in patients with taste disorders. Second, since the method requires patients to memorize different tastes, preservation of the cognitive function is essential. Third, differences based on gender, age, and race have not been investigated. Fourth, we did not examine the long-term effects of the training or how long the effect of gustatory sensitization might last. Fifth, it is unclear whether similar results can be obtained for all raw materials. While olfaction can identify an object from an odor, taste perceives the tastes of various raw materials as similar taste qualities. The reagents used in this study were all prepared from a single raw material, and similar results may not be obtained for different raw materials. In the future, similar training methods should be tried with a variety of raw materials. Finally, the results of this study showed improvement in taste recognition thresholds in participants exposed to single taste qualities. As meal consumption involves a complex interaction between various kinds of tastes, future studies should also focus on real food experience and examine training methods to evaluate the possibility of taste recognition in participants exposed to multiple taste qualities simultaneously.

Although the target population of the present study mostly comprised of young people, taste disorders emerge in a wide spectrum of diseases that may affect both young and older people due to their heterogeneous etiology. In addition to increasing the number of participants, future research should examine the differences in the effect of training based on factors such as age, gender, and underlying diseases. As taste sensitivity was found to increase with each additional training session, it will be necessary, in the future, to determine the extent of these changes in taste sensitivity induced by the training and how long its effects may last.

## Methods

### Target population

Recruitment was conducted by placing posters within the university campus and posting an application form on the Toho University School of Medicine website. The participants of the study were healthy adults aged between 20 and 64 years, who reported no taste abnormalities. All participants who enrolled in the study were given a written explanation about the content of the experiment, and informed consent was obtained prior to the commencement of the study. After an assessment of their oral condition, the participants were randomly assigned to two groups: a training group that received taste recall training to improve taste perception and a control group that did not receive training. Both groups were evaluated for taste sensitivity and the training group underwent taste recall training using filter paper discs impregnated with reagents for qualitative and quantitative taste analyses. Taste sensitivity evaluation and taste recall training were conducted in a speech and hearing training room, closed to any external sounds and smells to focus entirely on taste while controlling for any extraneous variables.

The study was conducted in accordance with the Declaration of Helsinki and other important ethical principles. This study has been approved by the Ethics Committee of Toho University School of Medicine (authorization no. A21024_A19032). This study was registered with the Japan Registry of Clinical Trials (jRCT1032210332) on 22/09/2021 and the data were made publicly available.

### Randomization

Participants were assigned to the training group and the control group using simple randomization. Participants were identified by their initials and date of birth and assigned a participant number. They were assigned a unique participant number by the investigator as per the order of a computer-generated randomized list.

### Oral status assessment

The participants’ oral status was assessed using the Oral Health Assessment Tool Japanese version (OHAT-J)^29^, and dental status was assessed using the Eichner classification^30^. Oral dryness was evaluated using an oral moisture checking device (Oral Moisture Checker Mucus^®^, Life Co., Ltd., Saitama)^31^. The measurement principle of this device is based on the impedance value generated through the tip of a capacitive sensor using the resonance frequency of the alternating current. The relative values reflecting the moisture content, were measured by applying the sensor to the tongue and the measurement value was determined to be the mean value of three consecutive measurements. A normal level of moisture content was 29.6 or higher, whereas a measurement value ranging from 28.0 to 29.5 was defined as borderline dry, and a value of 27.9 or lower indicated dryness of the oral cavity. Measurements were taken by pressing the oral moisture checker against the center of the lingual mucosa, approximately 10 mm from the tip of the tongue. Tongue pressure was measured using a tongue pressure measuring device (JMS Tongue Pressure Gauge^®^, JMS Co., Ltd., Hiroshima) to assess the participants’ oral motor skills^32^. After automatically adjusting the initial pressure of the probe’s balloon, participants were instructed to place the oral probe inside their mouth and bite the hard ring connected to it, to make sure that the probe did not shift. Then, they were asked to push the probe’s balloon against the hard palate with the tip of the tongue with maximum force and the tongue pressure was recorded. We considered a pressure of < 20 kPa as indicative of the risk of aspiration.

### Taste sensitivity assessment

Taste sensitivity was evaluated using 5 mm filter paper discs impregnated with reagents for qualitative and quantitative taste analyses (Taste Disc^®^, Sanwa Kagaku Kenkyusho Co., Ltd., Nagoya). The reagents consisted of test solutions for primary taste qualities (sweetness, saltiness, sourness, and bitterness), each having five different concentration levels (1–5), with 1 being the lowest and 5 being the highest. The substances used to represent sweetness, saltiness, sourness, and bitterness were sucrose, NaCl, tartaric acid, and quinine HCl, respectively. The concentration levels used for each substance were: 0.3%, 2.5%, 10%, 20%, and 80% for sucrose; 0.3%, 1.25%, 5%, 10%, and 20% for NaCl; 0.02%, 0.2%, 2%, 4%, and 8% for tartaric acid; and 0.001%, 0.02%, 0.1%, 0.5%, and 4% for quinine HCl. The filter paper discs were soaked in one of the four different taste solutions, randomly selected and applied, with pincers, on the participants’ tongues at increasing concentration grades starting from the lowest (1). The discs were placed 2 cm to the left of the tip of the tongue in the area innervated by the chorda tympani nerve for 2 s. The participants were asked to guess each taste they were exposed to, and the lowest concentration at which a specific taste was identified was noted as their taste recognition threshold. Before evaluating a new taste solution, participants were asked to thoroughly rinse their mouths to neutralize any lingering taste from the previous solution.

### Taste recall training

**Figure 3:**
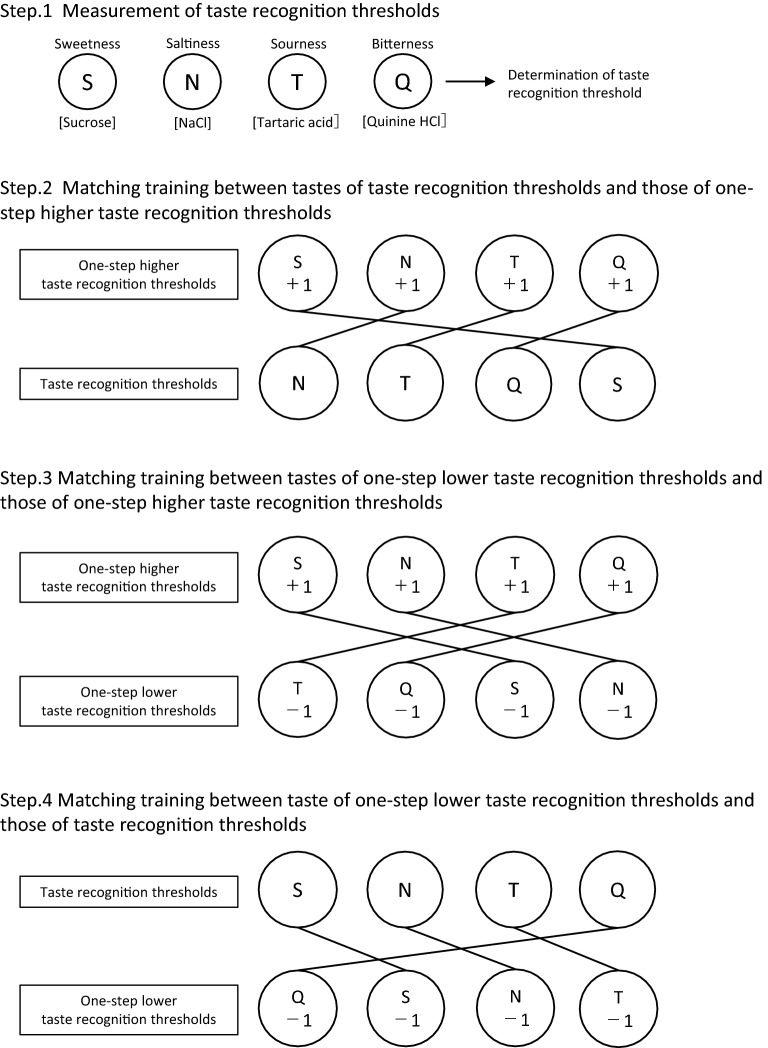
Overview of the taste recall training program. Filter paper discs impregnated with reagents for taste qualitative and quantitative analysis were used in all steps. S: sweetness, N: saltiness, T: sourness, Q: bitterness. + 1 indicates a one-step higher concentration than the initially measured taste recognition threshold and − 1 indicates a one-step lower concentration than the initially measured taste recognition threshold.

The training group underwent taste recall training for three days. The taste recall training process is composed of the following four steps (Fig. 3).

*Step 1* Participants’ taste recognition threshold for each of the four tastes was measured using the filter paper disc method.

*Step 2* Using the filter paper disc method, the participants were repeatedly exposed to each of the four tastes randomly. After having been told which taste was which, they were exposed to a one-step higher concentration than their taste recognition threshold in each of the four tastes until they could accurately identify them. Next, the participants were again repeatedly exposed to all the tastes in a random order, in the concentrations that matched their taste recognition threshold. Subsequently, they were asked to compare these tastes with each of the four tastes at the one-step higher concentration until their perception of each taste at the selected concentrations was perfectly matched.

*Step 3* As in step 2, the participants were repeatedly exposed to each of the four tastes using the filter paper discs, in a random order, after having been told which taste was which, at a one-step higher concentration than their taste recognition threshold until they could accurately identify them. Next, they were repeatedly exposed to all the tastes at a one-step lower concentration than their taste recognition threshold until they could accurately identify them. Further, they were asked to compare these tastes with each taste at a one-step higher concentration until their perception of each taste at the selected concentrations was perfectly matched.

*Step 4* As in step 2 and 3, using the filter paper disc method, the participants were repeatedly exposed to each taste, in random order, after having been told which taste was which, until they could accurately identify all of the four tastes at the taste recognition threshold. Next, they were repeatedly exposed to each of the tastes at a one-step lower concentration than their taste recognition threshold until they could accurately identify them. Further, they were asked to compare these tastes with each of the tastes at the taste recognition threshold until their perception of each taste at the selected concentrations was perfectly matched. This was done so that they could eventually identify each taste at a one-step lower concentration than their taste recognition threshold.

As in the evaluation of taste sensitivity, the filter paper discs used for taste recall training were placed 2 cm to the left of the tip of the tongue in the area innervated by the chorda tympani nerve. We compared the initial and final taste recognition thresholds between the training and control groups to examine changes in taste sensitivity. In the training group, daily changes in the taste sensitivity for each taste were examined.

**Figure 4:**
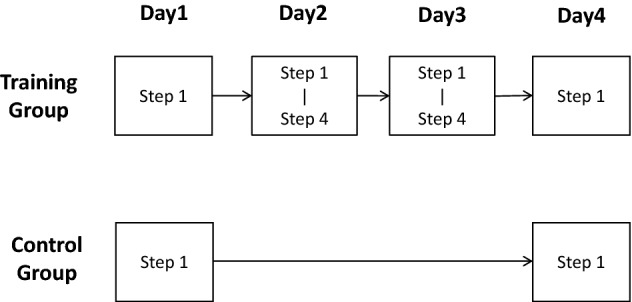
Flow chart of taste recall training program and taste recognition threshold assessment in the training and control groups.

The training group underwent taste recall training for 3 days, using filter paper discs impregnated with reagents for qualitative and quantitative taste analyses. On the fourth day, their taste sensitivity was evaluated again. Meanwhile, following the first evaluation, the participants in the control group went about their daily life as usual, and on the fourth day, their taste sensitivity was evaluated again (Fig. 4).

There were no changes to the trial outcomes or methods during the trials. Interim analyses were not conducted.

### Statistical analyses

The sample size was calculated based on the previous Taste Disc^®^ study^33^ with a significance level of 5%, a power of 80%, an effect size of 0.5, and a minimum requirement of 17 participants in each group.

Statistical analyses were conducted using software R version 3.6.3. We employed a paired t-test to analyze the changes between the initial and final taste recognition thresholds and a Friedman test to analyze daily changes in taste sensitivity of the training group. The significance level was set to 5% (two-tailed).

## Data Availability

The data that support the findings of this study are available from the corresponding author upon reasonable request.
